# Secondary seed removal in a degraded forest habitat in Madagascar

**DOI:** 10.1038/s41598-021-96306-7

**Published:** 2021-08-19

**Authors:** Paula M. A. Fiedler, Alice De Lapparent, Jeremie Razafitsalama, Justin Sanamo, Kim J. E. Steffens, Jörg U. Ganzhorn

**Affiliations:** 1 Institute of Zoology, Animal Ecology and Conservation, Universität Hamburg, Martin-Luther-King Platz 3, 20146 Hamburg, Germany; 2 Département de Biologie, Ecole Normale Supérieure, 75005 Paris, France; 3 Missouri Botanical Garden Antsiranana, BP 268, 201 Antsiranana, Madagascar; 4 Département Sciences de la Nature et de l’Environnement, Faculté des Sciences, Université d’Antsiranana, 201 Antsiranana, Madagascar; 5 Present Address: Département de Biologie, M2 Agroécologie, Connaissances, Territoires et Sociétés (ACTES), Université de Paris-Saclay, Paris, France

**Keywords:** Ecology, Zoology, Environmental sciences

## Abstract

Forest restoration is a prime goal within the 2021–2030 UN “Decade of Ecosystem Resoration”. As part of these activities, natural regeneration has to be promoted for biological as well as for economic reasons. For this, the processes of seed dispersal, seed predation and germination have to be understood in the original as well as in degraded vegetation formations. We used seed removal experiments to assess post-dispersal processes that influence recruitment along a gradient of forest degradation in Madagascar analyzing seeds of three animal dispersed tree species. The percentage of seeds consumed or dispersed, declined from forest (28.6%) to degraded forest (17.2%) to savanna (10.8%). Only three out of 1080 seeds were cached and remained intact during the 14-day experiment. All three seeds were cached in the forest habitat and none in the degraded forest and savanna. The low percentage of seeds removed may be due to the lack of endemic rodents caching seeds, as only introduced rats were recorded in the area. The species-poor fauna of potential secondary seed dispersers of the region and especially in the degraded areas might represent an obstacle for diverse regeneration in degraded regions of Madagascar.

## Introduction

Restoration of degraded land is a prime goal of the United Nations’ “Decade of Ecosystem Restoration” from 2021 to 2030^1^. Within this decade, planting trees is one of the main components of the African Forest Landscape Restoration Initiative (AFR100). Yet, planting trees is expensive and reforestation is all too often based on a few tree species of known properties that will result in species-poor forested landscapes. If areas to be reforested border remnants of natural forests, wind-dispersed seeds are more likely to arrive outside the forest than seeds dispersed by animals^2^. Yet, tree species with animal dispersed seeds dominate in natural tropical forests^3–5^. Thus, natural regeneration could be fostered by attracting seed dispersing animals into the areas to be restored. By depositing seeds of native plants in the reforestation area, animals help to diversify plant species in the regenerating cohort, thus facilitating regeneration towards systems with closer similarity to the original forest composition and reducing costs compared to planting forests of similar diversity^6–10^.

Regeneration can be compromised by seed predation or be modified by secondary dispersal in complex interactions^11–14^. While these interactions have been studied intensively within ecosystems, few studies have addressed the question on how natural forest regeneration is affected by secondary seed dispersal or seed predation on fallow land or in degraded forests targeted for forest restoration^15–21^.

A better understanding of the processes involved in forest restoration and regeneration and possible ways to achieve the restoration goals efficiently but at low costs is relevant for countries with limited resources. Madagascar is a special case, as most of the islands plants and animals are endemic and community composition of consumers differs from other parts of the world, such as Madagascar having a depauperate community of frugivorous species^22,23^. Therefore it is questionable, whether or not experiences from other parts of the world can be transferred to Malagasy systems without adaptations. At the same time, forest restoration is needed urgently, as this biodiversity hotspots has suffered from excessive habitat destruction, having led to a degree of fragmentation that makes the survival of a large proportion of the endemic biota questionable due to the small size of isolated populations^24–32^.

In this study, we describe an experimental study on secondary seed dispersal, seed predation, and fruit dispersal syndromes of tree species regenerating in a natural forest at different states of degradation as well as in the adjacent savanna in northern Madagascar. We use two native and one introduced tree species with fruits consumed by native animals and people alike, thus being of value not just for Madagascar’s endemic fauna but also for the local human population that uses not only the fruits but also the wood of these tree species for charcoal making^33–35^. Restoring forests with multi-use species increases the value of these forests for people and thus increases acceptance of reforestation initiatives. Thus, these three tree species fulfill the requirements of being dispersed by primary seed dispersers and their regeneration would contribute to the endeavor of ecosystem restoration to the benefit of people and Madagascar’s biota alike. Specific questions were: (1) does tree regeneration differ between habitat types; (2) does post-dispersal seed removal differ between parts of the forest at different states of degradation and the savanna habitat; (3) was seed removal due to secondary dispersal or seed predation.

## Results

### Seed fates

By the end of the experiment (after 14 days), none of the seeds of *Sclerocarya birrea* had been manipulated or removed by animals at any of the sites. Seeds of the other two species were recovered either at the place where they had been deposited at the beginning of the experiment or in the vicinity. Some seeds could not be recovered despite extensive searching. These seeds were classified as removed and eaten.

**Figure 1:**
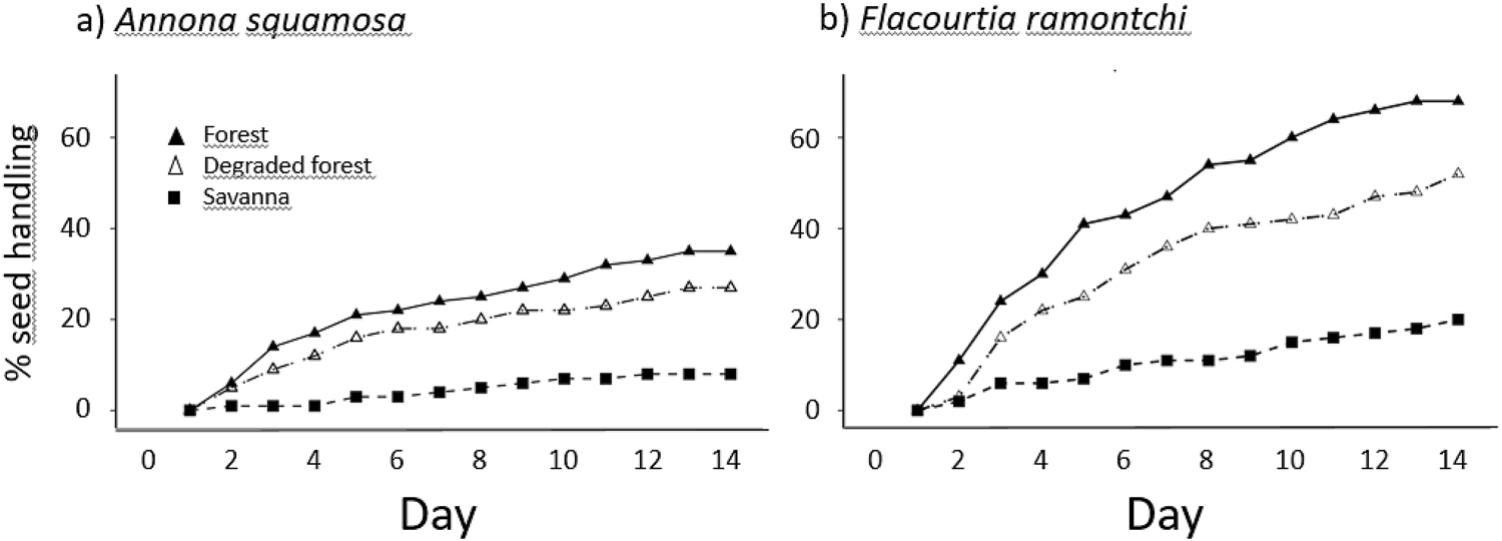
Cumulative percentage of seeds handled (i.e. eaten or removed) over time for (**a**) *Annona squamosa*, (**b**) *Flacourtia ramontchi* in the three different vegetation formations.

Seed removal from the experimental plots continued over the whole 14-day period of seed exposure. Removal was highest in the Forest and lowest in the Savanna. The highest proportion of seeds were removed during the first nights (Fig. 1; Table 1). Without considering *Sclerocarya birrea*, 42.9% of the seeds had been manipulated or removed by animals in the Forest, 25.8% in the Degraded forest and 16.8% in the Savanna. Of the seeds removed from the experimental plots, only three seeds survived until the end of the 14-day period buried in the Forest habitat. None of the seeds classified as handled in the Degraded forest and in the Savanna survived undamaged.

**Table 1:** Fates of thread-marked seeds in three vegetation formations of Oronjia, Madagascar. Since none of the seeds of *Sclerocarya birrea* were handled by animals, the results are also presented without this species.

	Seed fate					
	Not handled	Handled				Total
		Not removed	Removed			
			Not cached	Cached		
		Eaten at the seed station	Eaten away from the seed station	Eaten by the end of the study	Not eaten by the end of the study	
**Forest**						
Total count	257	52	44 (41)	4	3	360
% of total	71.4	14.4	10.8	1.1	0.8	
% without *S. birrea*	57.1	21.7	18.3 (17.1)	1.7	1.3	
**Deg. forest**						
Total count	298	42	20 (19)	0	0	360
% of total	82.8	11.7	5.6 (5.3)	0	0	
% without *S. birrea*	74.2	17.5	8.3 (7.9)	0	0	
**Savanna**						
Total count	321	16	23 (23)	0	0	360
% of total	89.2	4.4	6.4 (6.4)	0	0	
% without *S. birrea*	83.8	6.7	9.6	0	0	

Values in brackets are seeds that had disappeared and were not found again and classified as “eaten”.

**Figure 2:**
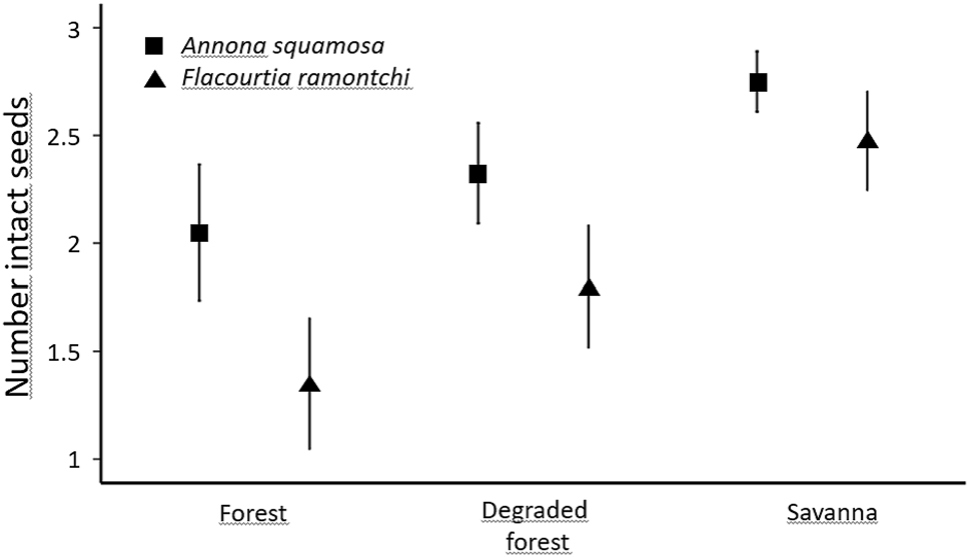
Average number of *Annona squamosa* and *Flacourtia ramontchi* seeds left intact at the experimental plots (N = 40 per vegetation formation) after 14 days. Though the data deviate from normality, values are means and 95% confidence intervals to illustrate differences between groups better than it would be possible with medians and quartiles.

When using the individual plots as the units for analyses, the number of seeds left intact at the seed plots differed between vegetation formations for *Annona squamosa* (Kruskal Wallis analysis of variance: H = 13.53, *p* < 0.001, *df* = 2) as well as for *Flacourtia ramontchi* (H = 27.67, *p* < 0.001, *df* = 2). The number of seeds left intact differed between the two species in the Forest and in the Degraded forest (Mann–Whitney U-test: z = 3.08 and z = 2.69, respectively; both *p* < 0.05 Bonferroni corrected; N = 40 plots per vegetation formation; Fig. 2). There was no difference between the two species in the Savanna (U = 1.70; *p* > 0.05; Fig. 2). *Sclerocarya birrea* is not considered as no seeds of this species had been manipulated by animals in the plots.

### Regeneration

We identified 58 different woody plant species with a diameter below 5 cm in the 36 2 × 2 m^2^ plots, 48 species in the Forest, 16 in the Degraded forest, and 9 in the Savanna. The number of regenerating species per plot was highest in the Forest with a median of 6 species per plot. The medians for the Degraded forest and the Savanna were 3 plant species per plot for both vegetation formations. The number of regenerating species per plot was significantly higher in the Forest than in the other two vegetation formations (Mann–Whitney-U: z > 3.35, *p* < 0.01 for both comparisons; Bonferroni corrected; N = 12 plots per vegetation formation). The numbers did not differ between Degraded forest and Savanna (z = 0.66, *p* = 0.5).

Regeneration varied with 1–19 plants with a stem diameter below 5 cm per 2 × 2 m^2^ plot. There were three outliers with 27 and 174 plants per 2 × 2 m^2^ plot in the Forest and one plot with 40 plants in the Savanna. The median numbers of woody plant individuals per 2 × 2 m^2^ plot for the Forest, Degraded forest and Savanna were 12.0, 5.5 and 11.0 individuals, respectively. Only the Forest plots differed significantly from the plots in the Degraded forest (Mann–Whitney-U: z = 3.71, *p* < 0.01). None of the other pairwise comparisons was significant.

**Figure 3:**
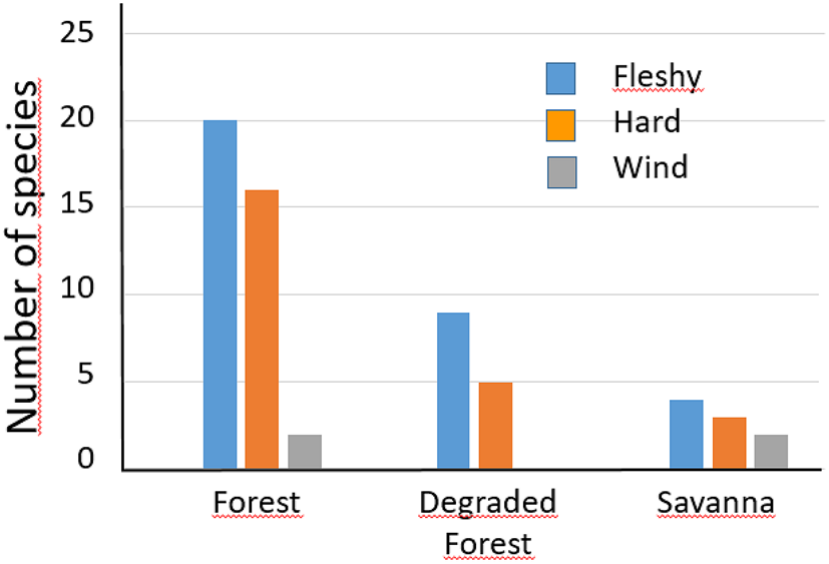
Representation of plant species with different types of fruit and seed dispersal in different vegetation formations.

In the regeneration plots, the Forest had about 20 woody species with fruits dispersed by frugivores and 2 woody species that are wind-dispersed. In the Savanna, the representation of woody species dispersed by animals or wind was four and two species, respectively (Fig. 3). The representation of plants with different dispersal syndromes (fleshy or arillate fruits or seeds for dispersal by vertebrates, hard fruits, winged for wind dispersal) in the regeneration plots did not differ between vegetation formations (chi-square = 4.08, *df* = 4, *p* = 0.42).

### Small mammal survey

We compiled a total of 400 trap-nights, 160 trap-nights in the Forest and Degraded forest and 80 trap-nights in the Savanna. In the Savanna we could trap only along one of the transects because traps disappeared quickly at the second transect. We did not catch any native rodent species but only one introduced *Rattus rattus* as possible seed predators in the Degraded forest and eight rats in the Savanna. *Setifer setosus* is primarily insectivorous and cannot damage or swallow seeds of the size used in our study. The Sherman traps as well as the cameras recorded *Setifer setosus* in the Forest and the Degraded forest habitat. The cameras did not provide any additional information relevant for the biodiversity inventory or the regeneration study.

### Grazing

Grazing pressure by cattle was highest on one transect in the Degraded forest and lowest on one transect in the Savanna. Goat fecal pellets were mostly restricted to the savanna that is closer to the village than the other vegetation formations (Table 2). The number of regenerating plants per 2 × 2 m^2^ plot decreases with increasing number of feces along the transect, though this relationship is not significant due to small sample size (Pearson correlation r = − 0.79, *p* = 0.06, n = 6).

**Table 2:** Livestock feces recorded along two transects and median number of regenerating trees < 5 cm in diameter per 2 × 2 m^2^ vegetation plot in three different vegetation formations in Oronjia, Madagascar.

	Forest	Degraded forest	Savanna
Feces: Cattle	20/27	57/25	12/27
Feces: Goats	0/1	0/1	15/22
Feces: Total	20/28	57/26	27/49
Median number of regenerating trees < 5 cm in diameter	12.5/11.5	5/7	12/7.5

The values for the two transects are separated by “/”.

## Discussion

In the forest habitat of northern Madagascar, the number of woody species in the regenerating cohort decreased with increasing forest degradation. In contrast, the number of woody plant individuals was similar in the forest habitat and in the savanna but lower in the degraded forest. Judging from the presence of livestock fecal samples, the degraded forest seemed to suffer most from livestock grazing, possibly resulting in the low number of plants in the regenerating cohorts in the degraded forest. Removal of seeds from the experimental plots also declined with degradation of the habitat. Seed removal resulted in seed predation but to a low percentage (< 1%) also in caching seeds intact and thus contributing to forest regeneration. The latter occurred only in the least degraded habitat type. The higher proportion of seeds removed in forest than non-forest habitat matches the results in other parts of the tropics, such as in Madagascar^16,36^, Asia^20^, South America^37^, and Africa^13^, but are inconsistent with data from Australia^38^ or other parts of the Americas^18,39^.

Compared to other sites, the proportion of seeds removed in Oronjia is very low, even in the least disturbed habitat. This may be due to the rarity of native rodents that would cache seeds. Our own small mammal survey was not extensive enough to document the whole small mammal community of the site. But more extensive surveys to the east at the Montagne des Français^40^ and the west at the Montagne d’Ambre^41,42^ revealed very low capture rates of *Eliurus* spp., probably the only native rodent species of the region that acts as secondary disperser and caches seeds^43^. In addition to obviously low abundance of this genus in the region, *Eliurus* spp. seem very sensitive to forest degradation^32,44,45^, thus limiting its role as seed disperser in degraded habitats.

Analyzing general patterns of forest regeneration in native forests as well as anthropogenic landscapes^46^ ought to consider a multitude of possible factors, such as the community composition of frugivores, primary and secondary seed dispersers and seed predators^12,36^, seed size and seed chemistry^38,47^, soil conditions and other ambient characteristics^13,37^. The lack of secondary dispersal and predation of *Sclerocarya birrea* seeds was unexpected. There are other examples that rodents do not consume seeds that are too large to be carried away and consumed away from the site where the seeds had been deposited, though size is not the only factor that determines seed dispersal^36,39,47–49^. Once germinated, seedlings face other constraints, such as competition, drought, fire or grazing^50–54^ to name but a few. In our case, the prevailing factor may be livestock grazing (Table 2) as it had also been identified in other parts of Madagascar while fire was recorded only once between 2006 and 2016 in Oronjia^55^.

Though the establishment of any given plant is influenced by many stochastic events, one of the first requirements for many plant species seems to be that their seeds are protected from desiccation, hoarded in a suitable place for germination, and hopefully forgotten by the hoarding animal^18^. Despite high seed predation levels, there are always some seeds cached intact or are still able to germinate despite having been partially eaten^56,57^. In the present study, only three out of 204 seeds handled by secondary seed dispersers and predators remained intact after the 14-day study period. Seed caching was only recorded in the forest environment. The proportion of surviving seeds may be low in this as well as in other studies^20,58^, but given the large number of seeds produced over the lifetime of a tree, even the low “escape rate” from seed predation is sufficient to guarantee survival of the lineage in most cases. Thus, high seed predation as measured by the removal of seeds from experimental plots, does not necessarily have to result in low regeneration. Rather, renaturation might be more successful with a species-rich community of seed eaters, including possible seed predators. Yet, degraded forest habitats and fallow land have a reduced community not only of primary seed dispersers, but also of secondary seed dispersers and seed predators that would hoard seeds and forget at least some of them that can then germinate^45,59–61^.

The present results may be relevant for the current efforts to restore forest habitats during the UN “Decade of Ecosystem Restoration”^1^. Within this endeavor, tree species diversity could be increased by facilitated restoration by luring primary seed dispersers into the area to be regenerated by planting fruit trees^7,34,35^. While this is certainly the first step necessary to bring seeds into the degraded landscape with different seed banks than the original forest^62,63^, it might be equally important to make sure that the habitat is also suitable for native secondary seed dispersers and seed predators, though, at first sight, this seems as counter-intuitive as promoting cattle and weedy shrubs for forest regeneration^57,64^. In a country like Madagascar that is famous for its unique flora and fauna, it is debatable whether non-native trees should be used for forest restoration^65^. Since people need forest resources, forest restoration has to take human needs into account in order to increase the value of a forest to the local people. For the time being, this is being done by using mostly exotic tree species as their growth properties and requirements are well known. But this can also be done by integrating many native and endemic tree species of value for animals and humans alike^34^. In either case it is important to know more about the processes involved in tree regeneration of native and exotic tree species to arrive at multi-use forests without promoting invasive species^66,67^.

## Methods

### Study area

The study was carried out in the Oronjia Protected Area (IUCN category V) in northern Madagascar, located between 12° 14′ 00.8″ S–12° 18′ 48.1″ S and 49° 22′ 44.8″ E–49° 23′ 34.0″ E between January and April 2019 by the end of the wet season. Average annual rainfall was around 1122 mm between 1981 and 2017, with 90% falling between November and April. Different anthropogenic pressures and military use have led to a degradation of Oronjia’s forest. Since 2007, conservation interventions were carried out in Oronjia, which culminated in the designation as a New Protected Area (“Nouvelle aire protégée”) in 2015. Today, the forest is in the process of regeneration, but the collection of yams and livestock breeding of cattle and goats remain^55,68^. Three habitat types were assessed within Oronjia: Forest, Degraded forest and Savanna. The three habitats were contiguous.

### Seed removal experiments

The experimental setup followed^20^ to facilitate further comparative analyses. In each habitat type, two transects of 475 m each were installed. Each transect was subdivided in 25 m intervals. Seed stations were established at each of the 25 m intervals along each of the transects, adding up to 20 seed stations (study points) per transect and 120 stations in total. At each seed station three seeds of each of the three plant species described below were deposited in a 1 m square located 1 m off the transect with 50 cm distance between seeds. Seeds of *Annona squamosa*, *Flacourtia ramontchi* and *Sclerocarya birrea* were used for the experiments. Seeds were extracted from ripe fruits collected directly from trees in case of *Annona squamosa* and *Flacourtia ramontchi* and from fallen fruits or bought from local people for *Sclerocarya birrea*. Fruit pulp was removed from the seeds. Mean seed sizes ranged from 6.4 × 5.2 mm in *Flacourtia ramontchi* to 28.3 × 20.0 mm in *Sclerocarya birrea* (Table 3). These tree species were chosen because of their economic value, because seeds of all three species are dispersed by native primary seed consumers, because seed size covered a wide range of sizes, and because seeds were available in sufficient quantities to complete the experiments in a standardized way. A 50 cm cotton thread was attached to each seed with non-toxic glue to facilitate the search for seeds removed from the experimental plots. Seed stations were checked daily for signs of feeding or insect infestation over a period of 14 days. Seeds that disappeared were searched for in the vicinity within a radius of about 5 m. Seeds were classified as eaten if seed fragments were found and cached if seeds were found below the soil surface or below leaf litter. Cached seeds were also monitored for the 14-day period. Seeds damaged by insects or rodents were classified as “eaten”.

**Table 3:** Mean seed sizes (with standard deviation) in mm; N = 10 for each species.

Species	Length	Diameter
*Annona squamosa*	14.5 ± 1.4	7.9 ± 0.7
*Flacourtia ramontchi*	6.4 ± 0.8	5.2 ± 0.4
*Sclerocarya birrea*	28.3 ± 0.9	20.0 ± 1.2

### Regeneration

At the study points 1, 4, 8, 12, 16 and 20 of each transect woody plants were identified and their size were recorded in a plot of 2 × 2 m^2^ (= 6 plots per transect). Identification was not possible for some of the seedlings. They were identified to the highest taxonomic unit or recorded as “unknown”. The circumference of each plant was measured before the first branching or at breast height. For plants with multiple stems, only the largest stem was measured. Circumferences were transformed into diameters. For the regeneration study only trees with a diameter < 5 cm were included.

Fruits were classified as “fleshy” (attracting fruigivores), “hard” (no external flesh but attractive for seed predators), “wind dispersed”. Fruit types were taken from the Generic Tree Flora of Madagascar^33^.

### Small mammal survey

Small mammals were trapped with Large Sherman Live Traps set in duplicates at each study point along the transects. Traps measured 7.6 × 8.9 × 22.9 cm and have been used in many standardized small mammal surveys in Madagascar and in the region (e.g.,^40,42^). At each study point, one Sherman trap was set on the ground and one was set up in trees or shrubs, resulting in 40 traps per transect. Traps were baited daily with peanut butter and banana before sunset and set for four consecutive nights in March and April 2019. Animals were identified, marked by hair clipping to be able to identify recaptures and released at the site of capture. In addition to the Sherman traps, five camera traps (Dörr Snapshot Wildlife Camera) were placed at the seed stations haphazardly. Cameras were only set in the Forest and Degraded forest because of the risk of theft in the open Savanna habitat.

### Grazing

Grazing pressure by goats and cattle was estimated by counting feces along the transects. Fecal remains estimated to be older than 48 h were not considered.

### Data analysis

For data deviating from normality, non-parametric tests were used as indicated in the Result section. For multiple pairwise comparisons, *p* values were Bonferroni corrected. Statistical analyses were run with SPSS (25.0). For the graphical illustration of differences between groups in analyses that were based on the individual plots, means and 95% confidence intervals were used instead of boxplots with medians, quartiles and ranges as they would be the appropriate graphical match for non-parametic statistical tests. This option was chosen because the number of intact seeds can vary between 0 and 3 per tree species and seed station. If we would plot standard box-whisker-plots, all six groups would cover the full range of values between 0 and 3, making visual comparisons difficult. In this case, confidence intervals allow easier visual comparisons of differences between groups than box-whisker-plots.

## References

[1] Gann GD (2019). International principles and standards for the practice of ecological restoration. Second edition. Restor. Ecol..

[2] Puerta-Piñero C, Muller-Landau HC, Calderón O, Wright SJ (2013). Seed arrival in tropical treefall gaps. Ecology.

[3] Howe HF, Smallwood J (1982). Ecology of seed dispersal. Annu. Rev. Ecol. Syst..

[4] Emer C (2020). Seed dispersal networks in tropical forest fragments: Area effects, remnant species, and interaction diversity. Biotropica.

[5] Simmons BI (2018). Moving from frugivory to seed dispersal: Incorporating the functional outcomes of interactions in plant-frugivore networks. J. Anim. Ecol..

[6] Holloway L, Lourenco W, Goodman SM (2000). Catalysing rainforest restoration in Madagascar. Biogeography of Madagascar.

[7] Styger E, Rakotoarimanana JEM, Rabevohitra R, Fernandes ECM (1999). Indigenous fruit trees of Madagascar: potential components of agroforestry systems to improve human nutrition and restore biological diversity. Agrofor. Syst..

[8] Crouzeilles R (2020). Achieving cost-effective landscape-scale forest restoration through targeted natural regeneration. Conserv. Lett..

[9] Chazdon RL (2014). Second Growth: The Promise of Tropical Forest Regeneration in an Age of Deforestation.

[10] Lamb D, Erskine PD, Parrotta JA (2005). Restoration of degraded tropical forest landscapes. Science.

[11] Boehning-Gaese K, Gaese BH, Rabemanantsoa SB (1999). Importance of primary and secondary seed dispersal in the Malagasy tree *Commiphora guillaumini*. Ecol..

[12] Boissier O, Feer F, Henry PY, Forget PM (2020). Modifications of the rain forest frugivore community are associated with reduced seed removal at the community level. Ecol. Appl..

[13] Aliyu B, Thia JA, Moltchanova E, Forget PM, Chapman HM (2018). Forest disturbance and seasonal food availability influence a conditional seed dispersal mutualism. Biotropica.

[14] Jordano P (2011). Frugivores and seed dispersal: mechanisms and consequences for biodiversity of a key ecological interaction. Biol. Lett..

[15] Nepstad DC (1999). Large-scale impoverishment of Amazonian forests by logging and fire. Nature.

[16] Dausmann KH, Glos J, Linsenmair KE, Ganzhorn JU (2008). Improved recruitment of a lemur-dispersed tree in Malagasy dry forests after the demise of vertebrates in forest fragments. Oecologia.

[17] Ostfeld RS, Manson RH, Canham CD (1997). Effects of rodents on survival of tree seeds and seedlings invading old fields. Ecology.

[18] Forget PM, Cuiljpers L (2008). Survival and scatterhoarding of frugivores-dispersed seeds as a function of forest disturbance. Biotropica.

[19] Guzmán CA, Howe HF, Wise DH, Coates RI, Zambrano J (2021). Rodent suppression of seedling establishment in tropical pasture. Oecologia.

[20] Blackham GV, Corlett RT (2015). Post-dispersal seed removal by ground-feeding rodents in tropical peatlands, Central Kalimantan, Indonesia. Sci. Rep.-Uk.

[21] Howe HF, Davlantes J (2017). Waxing and Waning of a Cotton Rat (Sigmodon toltecus) Monoculture in Early Tropical Restoration. Trop. Conserv. Sci..

[22] Donati G (2017). Low levels of fruit nitrogen as drivers for the evolution of Madagascar's primate communities. Sci. Rep.-Uk.

[23] Goodman SM, Benstead JP (2005). Updated estimates of biotic diversity and endemism for Madagascar. Oryx.

[24] Ganzhorn JU, Lowry PPI, Schatz GE, Sommer S (2001). The biodiversity of Madagascar:one of the hottest biodiversity hotspot on its way out. Oryx.

[25] Brinkmann K, Noromiarilanto F, Ratovonamana RY, Buerkert A (2014). Deforestation processes in south-western Madagascar over the past 40 years: what can we learn from settlement characteristics?. Agric. Ecosyst. Environ..

[26] Harper GJ, Steininger MK, Tucker CJ, Juhn D, Hawkins F (2007). Fifty years of deforestation and forest fragmentation in Madagascar. Environ. Conserv..

[27] Waeber PO, Wilmé L, Mercier J-R, Camara C, Lowry PP (2016). How effective have thirty years of internationally driven conservation and development efforts been in Madagascar?. PLoS ONE.

[28] Waeber PO (2015). Dry forests in Madagascar: neglected and under pressure. Int. For. Rev..

[29] Zinner D (2014). Analysis of deforestation patterns in the Central Menabe, Madagascar, between 1973 and 2010. Reg. Environ. Change.

[30] Vieilledent G (2018). Combining global tree cover loss data with historical national forest cover maps to look at six decades of deforestation and forest fragmentation in Madagascar. Biol. Cons..

[31] Ganzhorn JU, Rheinwald G (2000). Effects of fragmentation and assessing minimum viable populations of lemurs in Madagascar. Isolated Vertebrate Communities in the Tropics.

[32] Andriatsitohaina B (2020). Ecological fragmentation effects in mouse lemurs and small mammals in northwestern Madagascar. Am. J. Primatol..

[33] Schatz GE (2001). Generic Tree Flora of Madagascar.

[34] Konersmann C (2021). Using utilitarian plants for lemur conservation. Int. J. Primatol..

[35] Steffens KJE (2020). Lemur food plants as options for forest restoration in Madagascar. Restor. Ecol..

[36] Razafindratsima OH (2017). Post-dispersal seed removal by rodents in Ranomafana rain forest, Madagascar. J. Trop. Ecol..

[37] Aide TM, Cavelier J (1994). Barriers to lowland tropical forest restoration in the Sierra Nevada de Santa Marta, Colombia. Restor. Ecol..

[38] Osunkoya OO (1994). Postdispersal survivorship of north Queensland rainforest seeds and fruits: effects of forest, habitat and species. Aust. J. Ecol..

[39] Hammond DS (1995). Post-dispersal seed and seedling mortality of tropical dry forest trees after shifting agriculture, Chiapas, Mexico. J. Trop. Ecol..

[40] Sabel J (2009). The conservation status of mammals and avifauna in the Montagne des Français massif, Madagascar. Madagascar Conserv. Dev..

[41] Goodman SM, Andrianarimisa A, Olson LE, Soarimalala V (1996). Patterns of elevational distribution of birds and small mammals in the humid forests of Montagne d'Ambre, Madagascar. Ecotropica.

[42] Goodman SM, Ganzhorn JU, Olson LE, Pidgeon M, Soarimalala V (1997). Annual variation in species diversity and relative density of rodents and insectivores in the Parc National de la Montagne d'Ambre, Madagascar. Ecotropica.

[43] Goodman SM, Sterling EJ, Goodman SM (1996). The utilization of *Canarium* (Burseraceae) seeds by vertebrates in the RNI d'Andringitra, Madagascar. A floral and faunal inventory of the eastern side of the Réserve Naturelle Intégrale d'Andringitra, Madagascar: with reference to elevational variation.

[44] Ramanamanjato JB, Ganzhorn JU (2001). Effects of forest fragmentation, introduced *Rattus rattus* and the role of exotic tree plantations and secondary vegetation for the conservation of an endemic rodent and a small lemur in littoral forests of southeastern Madagascar. Anim. Cons..

[45] Ganzhorn JU (2003). Effects of introduced *Rattus rattus* on endemic small mammals in dry deciduous forest fragments of western Madagascar. Anim. Cons..

[46] Markl JS (2012). Meta-analysis of the effects of human disturbance on seed dispersal by animals. Cons. Biol..

[47] Yadok BG, Forget PM, Gerhard D, Aliyu B, Chapman H (2020). Seed nutrient content rather than size influences seed dispersal by scatterhoarding rodents in a West African montane forest. J. Trop. Ecol..

[48] Baraloto C, Forget PM (2007). Seed size, seedling morphology, and response to deep shade and damage in neotropical rain forest trees. Am. J. Bot..

[49] Yadok BG, Gerhard D, Forget PM, Chapman H (2018). Size doesn't matter: Larger Carapa seeds are not dispersed farther by African rodent community. Afr. J. Ecol..

[50] Ehrensperger T, Urech ZL, Rehnus M, Sorg JP (2013). Fire impact on the woody plant components of dry deciduous forest in Central Menabe, Madagascar. Appl. Veg. Sci..

[51] Ratovonamana YR, Rajeriarison C, Edmond R, Kiefer I, Ganzhorn JU, Beau N, Dessein S, Robbrecht E (2013). Impact of livestock grazing on forest structure, plant species composition and biomass in southwestern Madagascar. African Plant Diversity, Systematics and Sustainable Development—Proceedings of the XIXth AETFAT Congress, held at Antananarivo, Madagascar, 26–30 April 2010. Scripta Botanica Belgica.

[52] Pareliussen I, Olsson EGA, Armbruster WS (2006). Factors limitine the survival of native tree seedlings used in conservation efforts at the edges of forest fragments in upland Madagascar. Restor. Ecol..

[53] Manjaribe C, Frasier CL, Rakouth B, Louis EE (2013). Ecological restoration and reforestation of fragmented forests in Kianjavato. Int. J. Ecol..

[54] Randriamalala JR, Randriarimalala J, Herve D, Carriere SM (2019). Slow recovery of endangered xerophytic thickets vegetation after slash-and-burn cultivation in Madagascar. Biol. Cons..

[55] Goodman SM, Raherilalao MJ, Wohlhauser S (2018). Les aires protégées terrestres de Madagascar: leur histoire, description et biote/The terrestrial protected areas of Madagascar: their history, description, and biota.

[56] Wells K, Bagchi R (2005). Eat in or take away - Seed predation and removal by rats (muridae) during a fruiting event in a dipterocarp rainforest. Raffles B Zool.

[57] Paine CET, Beck H (2007). Seed predation by neotropical rain forest mammals increases diversity in seedling recruitment. Ecology.

[58] Van der Meer PJ, Kunne PLB, Brunsting AMH, Dibor LA, Jansen PA (2008). Evidence for scatter-hoarding in a tropical peat swamp forest in Malaysia. J. Trop. For. Sci..

[59] Gibson L (2013). Near-complete extinction of native small mammal fauna 25 years after forest fragmentation. Science.

[60] Herrera JP (2020). Effects of land use, habitat characteristics, and small mammal community composition on Leptospira prevalence in northeast Madagascar. PLos Neglect Trop. D.

[61] Irwin MT (2010). Patterns of species change in anthropogenically disturbed habitats of Madagascar. Biol. Cons..

[62] Valenta K, Steffens TS, Rafaliarison RR, Chapman CA, Lehman SM (2015). Seed banks in savanna, forest fragments, and continuous forest edges differ in a tropical dry forest in Madagascar. Biotropica.

[63] Randriamalala JR, Herve D, Letourmy P, Carriere SM (2015). Effects of slash-and-burn practices on soil seed banks in secondary forest successions in Madagascar. Agric. Ecosyst. Environ..

[64] Posada JM, Aide TM, Cavelier J (2000). Cattle and weedy shrubs as restoration tools of tropical montane rainforest. Restor. Ecol..

[65] Gérard A, Ganzhorn JU, Kull CA, Carrière SM (2015). Possible roles of introduced plants for native vertebrate conservation: the case of Madagascar. Restor. Ecol..

[66] Lavialle J (2015). Complementarity of native and introduced tree species: exploring timber supply on the east coast of Madagascar. Madagascar Conserv. Dev..

[67] Kull CA (2012). The introduced flora of Madagascar. Biol. Invasions.

[68] Missouri Botanical Garden. Plan d'aménagement et de gestion de la Nouvelle Aire Protégée Oronjia (Antananarivo, 2015).

